# High temperature *in-situ* observations of multi-segmented metal nanowires encapsulated within carbon nanotubes by *in-situ* filling technique

**DOI:** 10.1186/1556-276X-7-448

**Published:** 2012-08-08

**Authors:** Yasuhiko Hayashi, Tomoharu Tokunaga, Toru Iijima, Takuya Iwata, Golap Kalita, Masaki Tanemura, Katsuhiro Sasaki, Kotaro Kuroda

**Affiliations:** 1 Department of Frontier Materials, Nagoya Institute of Technology, Gokiso-cho, Showa-ku, Nagoya, 466-8555, Japan; 2 Department of Quantum Engineering, Nagoya University, Furo-cho Chikusa-ku, Nagoya, 464-8601, Japan

**Keywords:** Carbon nanotubes, *in-situ* filling method, Metal nanowires, Encapsulation, Transmission electron microscopy (TEM), Environmental TEM, Melting temperature

## Abstract

Multi-segmented one-dimensional metal nanowires were encapsulated within carbon nanotubes (CNTs) through in-situ filling technique during plasma-enhanced chemical vapor deposition process. Transmission electron microscopy (TEM) and environmental TEM were employed to characterize the as-prepared sample at room temperature and high temperature. The selected area electron diffractions revealed that the Pd_4_Si nanowire and face-centered-cubic Co nanowire on top of the Pd nanowire were encapsulated within the bottom and tip parts of the multiwall CNT, respectively. Although the strain-induced deformation of graphite walls was observed, the solid-state phases of Pd_4_Si and Co-Pd remain even at above their expected melting temperatures and up to 1,550 ± 50°C. Finally, the encapsulated metals were melted and flowed out from the tip of the CNT after 2 h at the same temperature due to the increase of internal pressure of the CNT.

## Background

Encapsulation of one-dimensional foreign materials into carbon nanotubes (CNTs) during CNT growth has received attention because they are expected to possess new physical and chemical properties based on CNT induced by nanospace [1,2]. Various metal nanowires have been successfully encapsulated within CNTs, employing mainly two kinds of methods. One of them is that CNTs are initially opened at their tube tips and subsequently filled with molten materials through capillary action [3-5]. The other one is an *in-situ* filling method, where the metals can be filled into the CNTs [6-8]. Among a variety of interesting applications, a promising application of ferromagnetic metal nanowires (such as Fe, Co, and Ni) encapsulated within a CNT is the high-density magnetic recording media due to their nanoscale size and strong anisotropic property, leading to small bit size [2-12]. Furthermore, the graphite layer provides an effective barrier against oxidation and consequently ensures a long-term stability of the metal inside CNTs [13].

Although it is very interesting to investigate a reaction process within the CNT due to the confined nanospace at various conditions, very few studies have been made so far on the detailed *in-situ* characterizations of the nanowire structure at various temperatures after encapsulation of the metal nanowire within CNTs.

Here, we present growth of self-assembled aligned Pd-Co-based multi-segmented one-dimensional metal nanowires encapsulated within multiwall CNT (MWCNT) arrays on Si by bias-enhanced microwave plasma chemical vapor deposition (MPCVD) with CH_4_ and H_2_. The metal nanowires encapsulated within MWCNTs were analyzed with transmission electron microscopy (TEM). Furthermore, *in-situ* microscopic environmental TEM (ETEM) was employed for *in-situ* observations of nanowires encapsulated within MWCNTs at a high temperature above melting points of metals.

## Methods

The Pd-Co-based nanowire encapsulated within MWCNTs was grown by bias-enhanced MPCVD using a 2.45-GHz, 1.5-kW microwave power supply, as described elsewhere [14]. A primary 6-nm-thick Pd metal thin layer and a secondary 9-nm-thick Co metal thin layers (Co/Pd: total thickness of 15 nm) were deposited on the thin barrier layer of SiO_2_ formed on the Si surface (Co/Pd/SiO_2_/Si substrate). The question arises why we chose Co/Pd bimetallic layers. Although we have previously reported Pd-based MF-CNTs using bias-enhanced MPCVD, we failed to fill the Co metal into the nanotubes using only a Co catalyst layer on the Si substrate. In combination with the Pd layer, we successfully encapsulated Co inside CNTs [14]. The feed gas, H_2_, was supplied into the plasma chamber to maintain a pressure of 20 Torr. The substrate was gradually heated up to 973 K by a radio-frequency graphite heater, and a microwave plasma was turned on to 600 kW. The Pd-Co-based nanowire within MWCNTs was grown for 15 min under a negative bias of 400 V at that maintained substrate temperature.

A JEOL (JEM-3010; JEOL Ltd., Akishima, Tokyo, Japan) TEM, operated at 300 kV, was used for room temperature observations. A Hitachi (H-9000NAR; Hitachi, Ltd., Minato, Tokyo, Japan) ETEM, operated at an accelerating voltage of 300 kV and equipped with a Gatan GIF and a Gatan CCD camera (Gatan, Inc., Pleasanton, CA, USA), was used for *in-situ* observations. A resistance-heating tungsten wire sample holder of a TEM was used to heat the nanowire encapsulated within MWCNTs up to 1,550°C in vacuum with an accuracy of ±50°C depending on the sample position [15].

## Results and discussion

**Figure 1:**
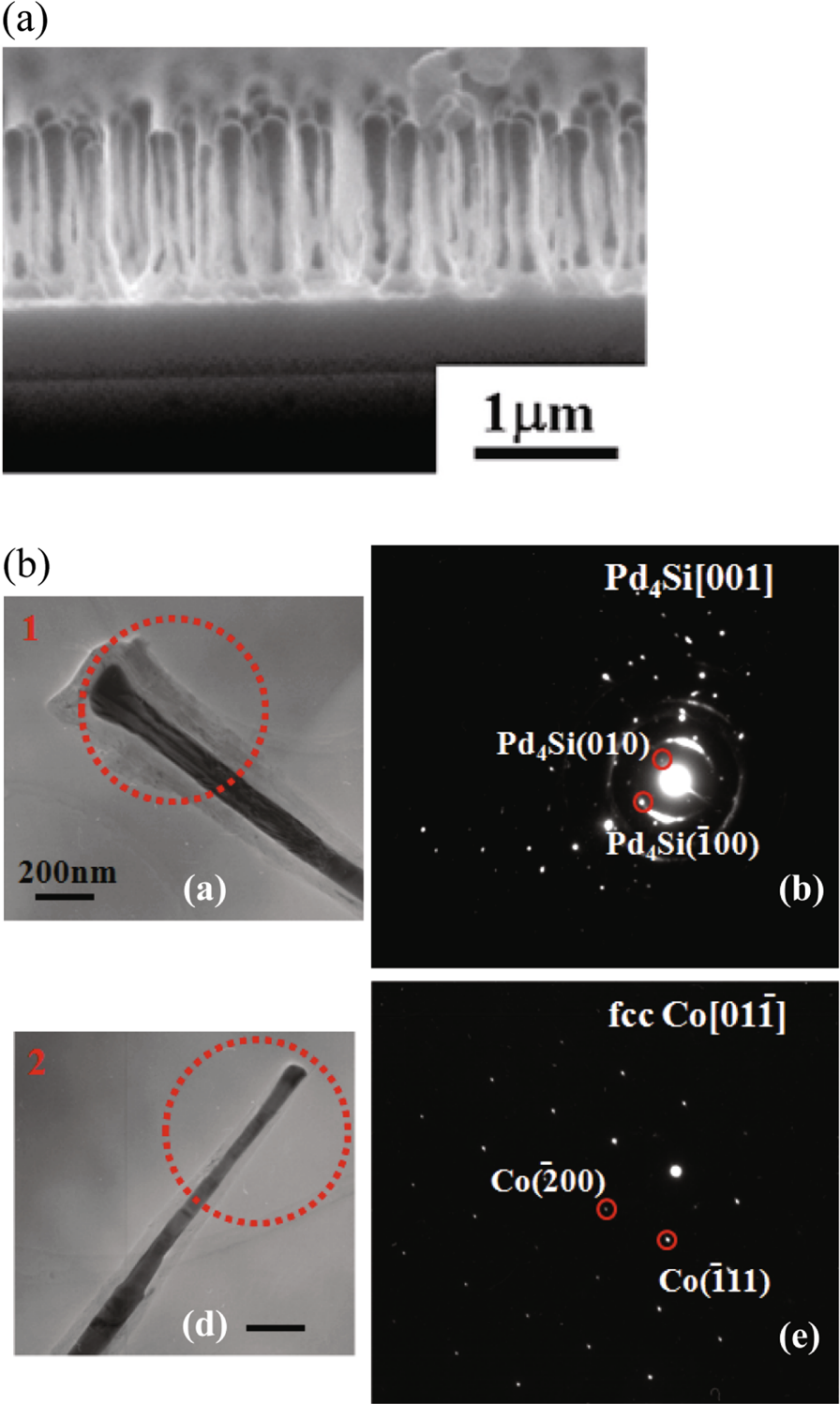
**SEM, TEM, and SAED.** (**a**) Cross-sectional SEM image. (**b**) TEM images and selected area electron diffractions observed at the bottom part (**a**, **b**) and top part (**d**, **e**) of the metal nanowire encapsulated within the MWCNT.

**Figure 2:**
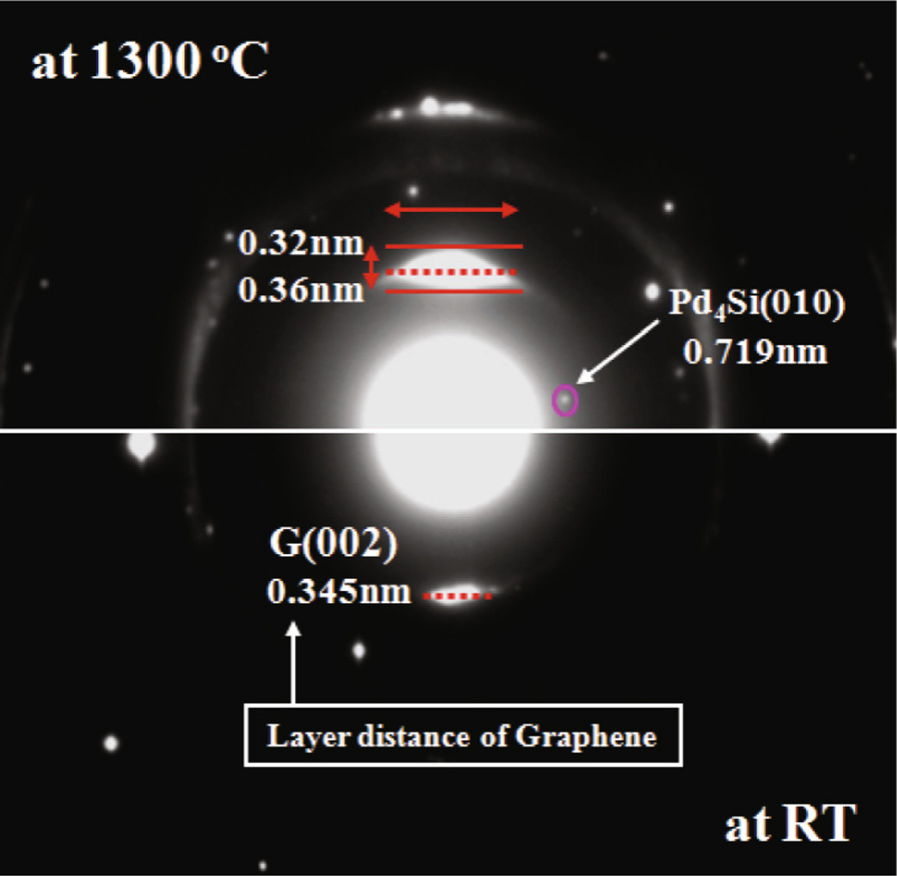
Selected area electron diffractions observed at room temperature and 1,300°C, respectively, by ETEM.

Figure 1a shows the scanning electron microscope (SEM) cross-section image of the typical sample for subsequent characterization via ETEM. Based on the SEM image, the wire- or rod-like blackish color of the metal inside the CNT tubes were clearly observed. Therefore, the encapsulation of the metal inside the CNTs has successfully occurred during CNT growth. Figure 1b shows the TEM images and electron diffraction patterns of the bottom and tip parts of the Pd-Co-based nanowire encapsulated within the MWCNT. We have highlighted the one-dimensional nanowire structure fully encapsulated within MWCNTs, found in the as-grown sample by TEM. Based on Figure 1a,d, it is noteworthy that the inner diameter of the MWCNTs is significantly reduced from the lower (near to the tube bottom, *ca.* 102 nm) to the upper sites (near to the tube top, *ca.* 55 nm). The diffraction patterns were determined as Pd_4_Si and Co nanowires at the bottom and top parts of the MWCNT, respectively. Details of the tube top part within the MWCNT will be discussed later. We have successfully demonstrated multi-segmented one-dimensional metal nanowires by template-free *in-situ* filling technique (see Figure 2a). Based on our previous study, the demixing in a solid-soluted Co-Pd alloy was induced by microwave plasma hydrogen irradiation at a relatively lower temperature. Then, these intermetallic compounds decomposed into Pd/Co by two phases when subjected to growth temperature at 973 K [16]. The catalyst particle was assumed to be composed of Co and Pd regions, where the Co region is onto the Pd region due to the interaction of Co or Pd metals with the SiO_2_ layer, which may provide a key clue to explain the nature of multi-segmented metal nanowires encapsulated within CNTs [17]. The Pd region is easier to react with the SiO_2_ layer, giving the Pd_4_Si, rather than Co region because of the order of Co and Pd regions on the SiO_2_/Si substrate. Although *in-situ* filling of Fe, Co, Ni, or Cu can also be achieved in chemical vapor deposition systems [7,18] by pyrolysis of organometallic compounds, most of the research so far has reported a single metal encapsulated within MWCNTs. The Co nanowire encapsulated within the MWCNT at the tip part was always present as the face-centered-cubic (fcc) Co structure based on selected area electron diffraction (SAED) measurements. The reaction between Pd and Si is more favorable than that between Co and Si, so that the dissolved Si from the substrate possibly caused the stoichiometric structure of Pd_4_Si and Si-incorporated Co during growth of MWCNT [19]. Moreover, it is difficult to form a stable palladium-carbide. The silicide formation of Pd_4_Si by reaction of Pd with hydrogenated amorphous silicon (*a*-Si:H) substrate and SiO_2_ substrate was reported so far [20,21]. The reduction of Pd/SiO_2_ catalysts in hydrogen irradiation at 973 K may already lead to a considerable interaction between palladium and silica [21].

Figure 2 shows the diffraction pattern observed at room temperature and 1,300°C. We clearly observed strain-induced deformation on the spot of graphite G(002). It is very interesting that the solid-state phases remain even at above melting points of Pd_4_Si (890°C) and Co-Pd (1,250°C). Based on the diffraction pattern, we estimated that the graphene layer distance varies between +0.015 and −0.025 nm at 1,300°C compared to that of RT. Therefore, both positive and negative fluctuations of interlayer spacing of graphene layers at several positions may appear to relax the residual thermal strain. The fluctuation of distance between graphene layers is metastable up to the formation of dislocations in graphene layers.

**Figure 3:**
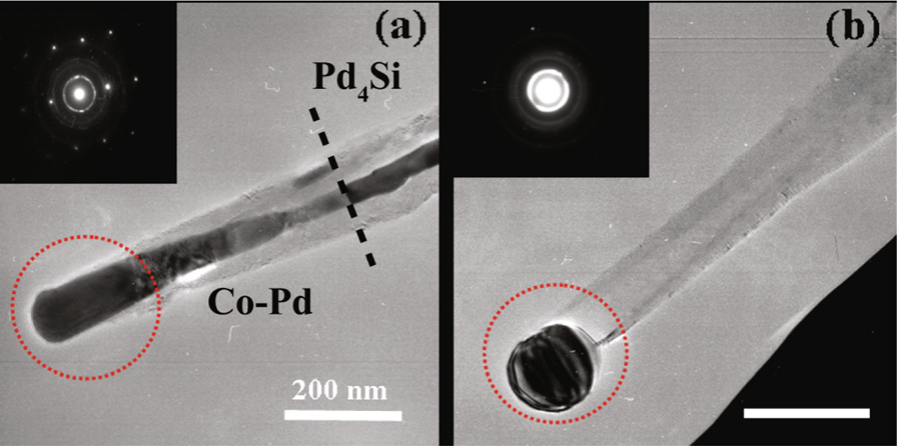
**TEM images and selected area electron diffractions.** Observed at (**a**) 1,550°C and (**b**) after 2 h at the same temperature by ETEM.

Figure 3a shows the TEM image and electron diffraction pattern observed at 1,550°C by using an ETEM. It is worth mentioning that the tube top region always encapsulated with the Pd-Co nanowire on top of the Pd_4_Si nanowire even at high temperature. The interface between Pd_4_Si and Pd-Co becomes blurred with increasing temperature. Ambiguity in the interface structure still exists and needs further investigation by high-resolution ETEM. The encapsulated metals were melted and flowed out from the tip of the CNT after maintaining the sample at the same temperature for 2 h, as shown in Figure 3b. Considering the setting temperature of 1,550°C (accuracy within ±50°C), the generated metallic sphere with a diameter of 143 ± 5 nm is presumably the molten phase based on electron diffraction pattern. The external diameters of the CNT before (Figure 3a) and after (Figure 3b) flowing out of the inner metal are approximately 120 and 95 nm, respectively. This indicates that the thermal expansion of internal metals causes the accumulation of internal pressure inside the CNT [22]. As a result, the CNT tip breaking occurred after a threshold internal pressure.

According to the experiments, the diffraction patterns indicate that both Pd_4_Si and Co-Pd have a crystalline structure even at the melting points. This may be due to the confined nanospace effect. Recently, Kobayashi et al. reported encapsulation of Sn, Pb, Ag, and Au within MWCNTs by capillary action. The results suggest that a confined nanospace prevents crystal growth of metals having a low melting point [23]. Confinement of metals within a nanospace still remains an interesting question for both theoretical research and industrial application.

## Conclusion

We synthesized multi-segmented one-dimensional metal nanowires within MWCNTs by *in-situ* filling technique during PECVD growth of MWCNTs. According to the TEM images and SAED of metal nanowires within the MWCNT, the Pd_4_Si nanowire and fcc Co nanowire on top of the Pd nanowire were encapsulated within the bottom and top parts of the MWCNT, respectively, by *in-situ* filling technique. The solid-state phases of Pd_4_Si and Co-Pd remain even at above their melting points at 890°C and 1,250°C, respectively, by ETEM. This may be due to the confined nanospace effect. We observed strain-induced deformation on the spot of graphite G(002) at 1,300°C. The accumulated internal pressure due to high temperature at 1,550°C caused the break of the CNT tip and pushed out the molten metal confirmed by electron diffraction pattern.

## Competing interests

The authors declare that they have no competing interests.

## Authors’ contributions

YH and TT designed the research. TI1 participated in the design of the sample preparation. TT and KS set the environmental condition of environmental TEM and carried out the environmental TEM. TI2, GK, MT, TY, and KK participated in the design and coordination of the research. All authors read and approved the final manuscript.
